# P-30. Thymosin-α1 for Sepsis Management: A Systematic Review and Meta-Analysis of 1972 Patients

**DOI:** 10.1093/ofid/ofaf695.259

**Published:** 2026-01-11

**Authors:** José Rafael Aguilar González, Alan Alejandro A Aparicio Osorio, Iojana Cruz Coba, José Luis Aranda García, Julio Ascención López, Rubén Bandala Vargas, Mauricio Aguilar González

**Affiliations:** Benemérita Universidad Autónoma de Puebla, Puebla, Puebla, Mexico; Benemerita Universidad Autónoma de Puebla, Puebla, Puebla, Mexico; Benemérita Universidad Autónoma de Puebla, Puebla, Puebla, Mexico; Benemértia Universidad Autónoma de Puebla, Azochiapan, Morelos, Mexico; Benemérita universidad autónoma de Puebla, Puebla, Puebla, Mexico; Universidad de la Salud del Estado de Puebla, Puebla, Puebla, Mexico; Oklahoma State University, Stillwater, Oklahoma

## Abstract

**Background:**

Sepsis remains a leading cause of death in critically ill patients, largely due to a dysregulated immune response that progresses to immunosuppression. Thymosin Alpha 1 (Tα1), an endogenous immunomodulatory peptide, has shown therapeutic potential by restoring immune function through T cell and dendritic cell activation and cytokine regulation. Recent studies suggest that its use as adjunctive therapy may reduce mortality and improve clinical outcomes. This work evaluates the role of Tα1 as an immunomodulatory strategy in the treatment of sepsis.

**Figure:**
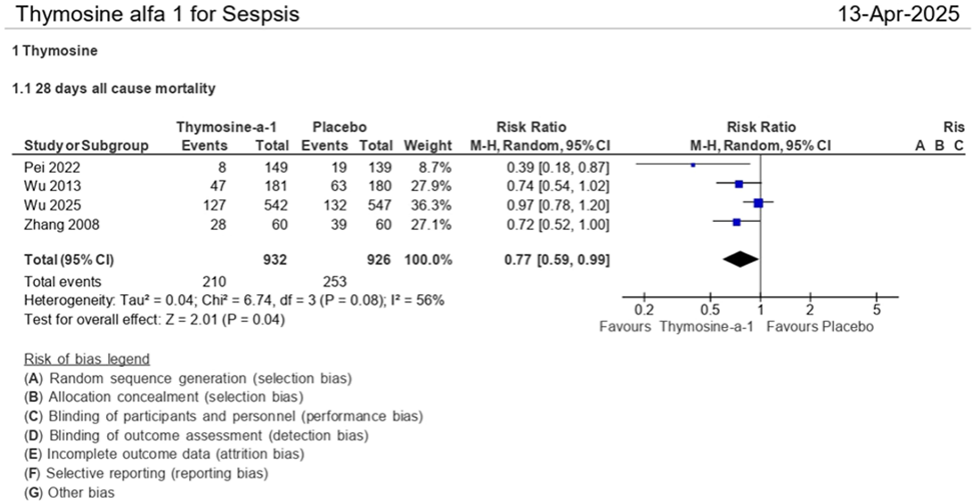
28 days all cause of death. Thymosin-α1 treatment significantly reduced 28-day all-cause mortality compared to placebo (RR = 0.77 [95% CI: 0.59–0.99], p = 0.04), suggesting a survival benefit in critically ill patients.

**Figure:**
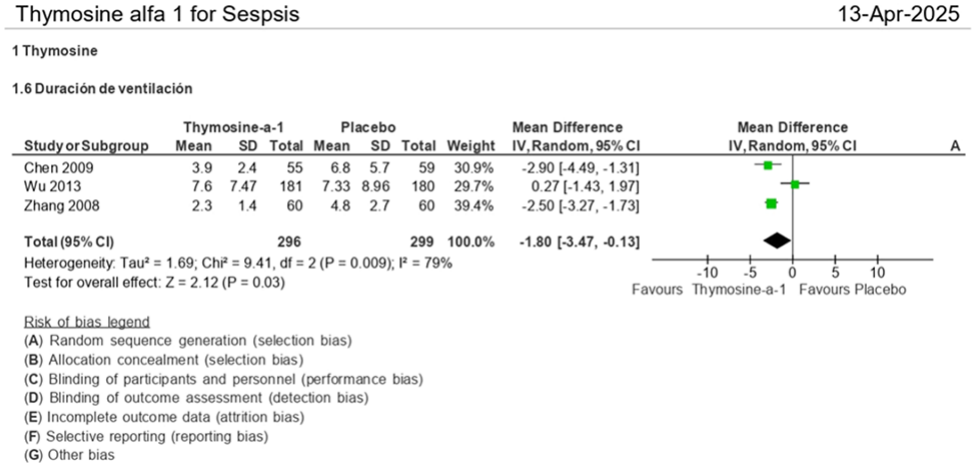
Duration of Mechanical Ventilation Patients receiving thymosin-α1 had a shorter duration of mechanical ventilation (mean difference = -1.80 days [95% CI: -3.47 to -0.13], p = 0.03), indicating faster respiratory recovery.

**Methods:**

A systematic search was performed in Pubmed, CENTRAL, OVID, Scopus, following PRISMA guidelines, including studies up to 7th March 2025. Measures of effect were relative risk and mean differences.

**Figure:**
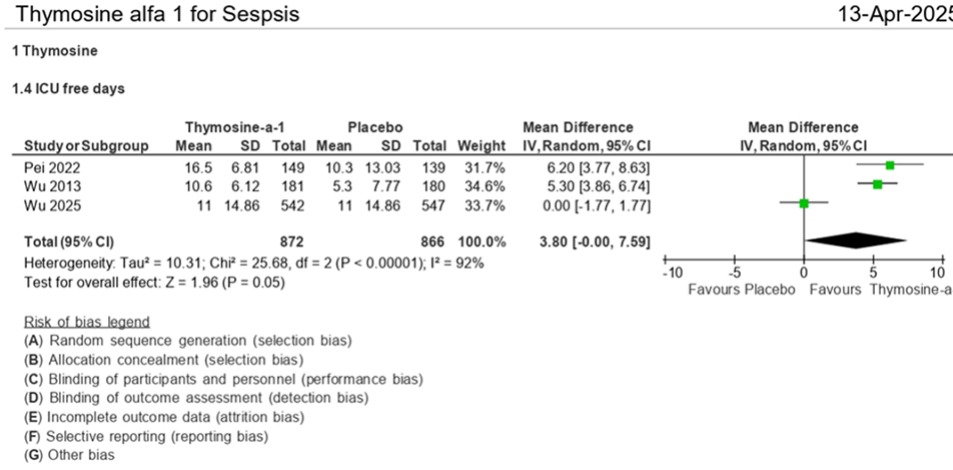
ICU-free days. Compared to placebo, thymosin-α1 was associated with more ICU-free days (mean difference = 3.80 days [95% CI: 0.00–7.59], p = 0.05), suggesting reduced ICU dependency.

**Figure:**
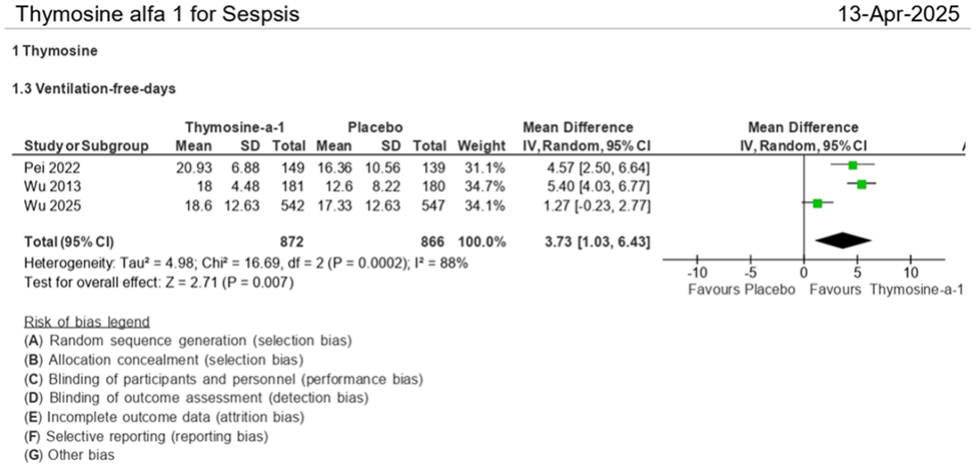
Ventilator-Free Days. Thymosin-α1 increased ventilator-free days (mean difference = 3.73 days [95% CI: 1.03–6.43], p = 0.007), reflecting improved respiratory independence.

**Results:**

Patients receiving thymosin-α1, compared to placebo, show a reduction in 28-day all-cause mortality (RR = 0.77 [95% CI: 0.59, 0.99], p = 0.04), length of mechanical ventilation (RR = -1.80 [95% CI: -3.47, -0.13], p = 0.03), ventilator-free days (RR = 3.73 [95% CI: 1.03, 6.43], p = 0.007), ICU-free days (RR = 3.80 [95% CI: 0.00, 7.59], p = 0.05), and ICU mortality mortality (RR = 0.76 (95% CI: 0.59, 0.98], p = 0.04). Although there is a trend toward shorter ICU stays in the thymosin group (RR = -2.49 (95% CI: -6.15, 1.16], p = 0.18), this difference is not statistically significant.

**Conclusion:**

Treatment with Thymosin alfa-1 in patients with sepsis demonstrated a significant reduction in both ICU and 28-day mortality, an increase in ICU- and mechanical ventilation-free days, and a shorter duration of supportive therapy. Although no significant decrease in total ICU length of stay was observed and some heterogeneity was present in the results, the data suggest that Thymosin alfa-1 may substantially improve clinical outcomes in patients with sepsis, further studies are neccesary to address the overall effectivness of thymosin alfa-1.

**Disclosures:**

All Authors: No reported disclosures

